# 

**Published:** 1826-12

**Authors:** 


### MONTHLY LIST OF MEDICAL BOOKS.

[*No books can be entered on this List except those sent to us for the purpose; as, in the list hitherto transmitted, the names of works have frequently been given as published, which have not appeared for weeks, or even months, after.*]

The Morbid Anatomy of the Human Brain: being Illustrations of the most frequent and important Organic Diseases to which that Viscus is subject. By Robert Hooper, M.D. Bachelor of Physic in the University of Oxford. With the Plates coloured.—4to. London, 1826.

Materia Indica; or, some Account of those Articles which are employed by the Hindoos, and other Eastern Nations, in their Medicine, Arts, and Agriculture: comprising also Formulæ, with practical Observations, Names of Diseases in various Eastern Languages, and a copious List of Oriental Books immediately connected with General Science, &c. &c. By Whitelaw Ainslie, M.D. M.R.A.S. late of the Medical Staff of Southern India. Vol. I.—London, 1826.

Lexicon Pharmacopœlium, or a Pharmacopœial Dictionary; containing the London Pharmacopœia of 1824, in Latin and English; the Chemical Decompositions; a Description of the Simples and Compounds of the Pharmacopœias of London, Edinburgh, and Dublin, with their Properties, Use, and Doses; and in which all former Names are referred to the present Pharmaceutical Nomenclature. To the whole is annexed an English Index of Technical and Domestic Terms relative to Medicines; a Table showing at one view the common and Linnæan Names; Dr. Cullen's and Mr. John Murray's Arrangement of the Materia Medica; a Dictionary of Operative Medicine; and, lastly, two serviceable Vocabularies for Translating Physicians' Prescriptions and the Pharmacopœias. Designed expressly for the Use of Students. By Thomas Castle, Member of the Physical Society, Guy's Hospital.—18mo. London, 1826.

Progress of the System for the effectual Removal of Impediments in Speech, discovered by JOHN BROSTER, F.A.S E. For the third Year.—1826.

The Use of the Chlorate of Soda, and the Chlorate of Lime. By A. G. LABARRAQUE, Pharmacien of Paris, &c. Translated by JAMES SCOTT, Surgeon.—8vo. London, 1826.

Obstetric Plate, No. I. This Plate represents a Fœtus in Utero, enveloped in the Membranes at the seven and a half Month of Gestation, with the Placenta attached to the Body, Cervix, and Os Uteri, as taken from the Subject thirty-six Hours after Death. By GEORGE JEWEL, Surgeon, Lecturer in Midwifery, &c.

☞ This Plate, which is drawn on stone, gives an extremely good representation of the parts, and is highly creditable to Mr. Jewel.

### METEOROLOGICAL JOURNAL,

### NOTICES.

*Communications have been received from* Mr. Professor Mackenzie, Dr. Robertson, Mr. Wood, Mr. Boyle, Mr. Anderson, Dr. MacAndrew, Mr. Kennedy, *and* Dr. Paul.

*We shall feel much obliged to* Mr. Mackenzie *for the Paper to which he alludes.*

*We shall be glad to hear further from* Mr. C. C.: *his Paper in an early Number.*

